# ITGB3 promotes cisplatin resistance in osteosarcoma tumors

**DOI:** 10.1002/cam4.5585

**Published:** 2023-02-11

**Authors:** Qian Li, Guangyou Chen, Huachai Jiang, Haoping Dai, Dongdong Li, Kai Zhu, Kaiquan Zhang, Huarui Shen, Houping Xu, Sen Li

**Affiliations:** ^1^ The Affiliated Traditional Chinese Medicine Hospital of Southwest Medical University Luzhou Sichuan P.R. China

**Keywords:** apoptosis, cisplatin, CRISPR/Cas9, ITGB3, osteosarcoma, transcriptome

## Abstract

**Objective:**

Osteosarcoma is the most malignant and common primary bone tumor with a high rate of recurrence that mainly occurs in children and young adults. Therefore, it is vital to facilitate the development of novel effective therapeutic means and improve the overall prognosis of osteosarcoma patients via a deeper understanding of the mechanisms of chemoresistance in osteosarcoma progression.

**Methods:**

In this research, the relationship between ITGB3 and the clinical characteristics of patients was detected through analysis of publicly available clinical datasets. The expression of ITGB3 was analysis in collected human osteosarcoma tissues. In addition, the potential functions of ITGB3 in the cisplatin resistance of osteosarcoma cells were investigated in vitro and in tumor xenotransplantation. Finally, the molecular mechanism of ITGB3 in the progression and recurrence of osteosarcoma were explored via transcriptome analysis.

**Results:**

ITGB3 was identified as a potential regulator of tumorigenicity and cisplatin resistance in relapsed osteosarcoma. Furthermore, the decreased osteosarcoma cell proliferation and migration ability in ITGB3 knockout osteosarcoma cells were related to increased apoptosis and slowing cell cycle progression. In addition, ITGB3 had a positive correlation with cisplatin resistance in cells and tumor xenografts in mice. Accordingly, ITGB3 performed the functions of proliferation and cisplatin resistance in osteosarcoma through the MAPK and VEGF signaling pathways.

**Conclusion:**

Our results will contribute to a better understanding of the function and mechanism of ITGB3 in osteosarcoma cisplatin resistance and provide a novel therapeutic target to decrease cisplatin resistance and tumor recurrence in osteosarcoma patients.

## Graphical abstract

In the present work, ITGB3 knockout osteosarcoma cells had the decreased proliferation and migration ability, increased apoptosis and slowing cell cycle progression in vitro, and reduced cisplatin resistance in vivo. Further analyses suggested that MAPK and VEGF signaling pathways may involved in these process.

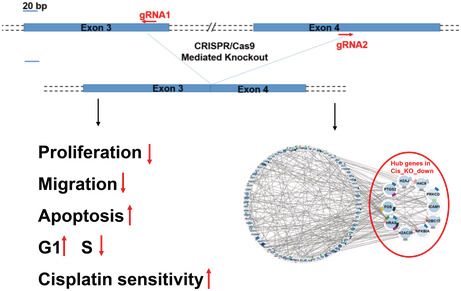

## 1 INTRODUCTION

Osteosarcoma is one of the most common primary and malignant bone tumors and mainly affects children and adolescents.^1^ The survival rate of osteosarcoma patients has been improved by treatment with a combination of surgery and perioperative neoadjuvant chemotherapy, in which cisplatin, doxorubicin, and methotrexate are commonly used chemotherapeutic drugs.^2^ However, in patients who respond poorly to these drugs, the rate has remained roughly constant over the past three decades.^3^, ^4^, ^5^ Even though many patients who use additional doses or drugs still experience distant metastasis and relapse, the five‐year survival rate is only approximately 20%.^6^, ^7^ Notably, poor prognosis is closely related to drug resistance. Therefore, to develop novel effective treatment means and dramatically improve osteosarcoma patient prognosis, it is imperative to explore the molecular mechanisms of osteosarcoma chemotherapy resistance.

β3 integrin (ITGB3), also called GP3A or CD61, is one of the most extensively studied members of the integrin family. ITGB3 plays diverse important functions in the progression of malignant tumors and the molecular reprogramming of the microenvironment.^8^ In recent years, some research has reported that there is a strong relationship between ITGB3 and drug resistance.^9^, ^10^, ^11^ Naik et al. indicated that the NRP1‐ITGB3 axis also mediates the chemoresistance response of breast cancer cells.^12^ Other evidence suggests that inhibiting ITGB3 expression increases the antitumor activity of ALK inhibitors in ALK‐rearranged non‐small cell lung cancer.^13^ The overexpression of ITGB3 is also involved in the resistance to EGFR inhibition, mechanistically due to the complex formed by ITGB3/KRAS/RalB and the activation of TBK1 and NFκB that the complex mediates.^14^, ^15^ However, the relationship between ITGB3 expression and clinical prognosis and recurrence in osteosarcoma remains unclear.

In this research, we detected the relationship between ITGB3 and the clinical characteristics of patients and analyzed the expression of ITGB3 in human osteosarcoma tissues. In addition, the potential functions of ITGB3 in the cisplatin resistance of osteosarcoma cells were investigated in vitro and in tumor xenotransplantation. Finally, we further explored the molecular mechanism of ITGB3 in the progression and recurrence of osteosarcoma.

## 2 MATERIALS AND METHODS

### 2.1 Patients and tissue samples

The raw count data of RNA‐sequencing (RNA‐Seq) and the relevant clinical information of 95 osteosarcoma samples in this study were obtained from the TARGET (Therapeutically Applicable Research to Generate Effective Treatments) dataset (https://ocg.cancer.gov/programs/target), in which the requirements and application procedures complied with relevant protocols and policies. For Kaplan–Meier (KM) curves, the survival analysis with *p* values, hazard ratio with 95% confidence interval, and log‐rank test were chosen to compare the difference of overall survival (OS) and disease‐free survival (DFS) between two groups: ITGB3 high and low‐expression groups (Table S1). Time‐series receiver operating characteristic analysis was performed to compare the predictive accuracy of the genes and their risk score. All R packages and analytical procedures were executed using R software (v4.0.3). Primary and recurrent osteosarcoma specimens assayed in this research were gathered from the *Department of Spinal Surgery*, *The Affiliated Traditional Chinese Medicine Hospital of Southwest Medical University* and informed consent was obtained from all study patients. This research was approved by the Ethics Committee. All procedures performed in this study were approved by the Ethics Committee of *The Affiliated Traditional Chinese Medicine Hospital of Southwest Medical University* (KY2022021) and followed the ethical standards and principles of the Helsinki Declaration and its later amendments.^16^

### 2.2 Cells and culture medium

The osteosarcoma cell lines (143B) were purchased from the *National Infrastructure of Cell Line Resource* (NICLR, China) and kept in our laboratory and were cultured in Dulbecco's modified Eagle's medium (Gibco) plus 10% fetal bovine serum (HyClone). The cell lines used in this research were cultured at 37°C with 5% CO_2_. When the cells occupied 80% confluence in the dish or plates, they were dissociated with 0.05% trypsin (Gibco) at 37°C for 3 min and harvested for passaging or used for subsequent experiments. The cell numbers were detected using a Rigel cytometer in this research (Countstar).

### 2.3 Cell counting kit‐8 assay

The doubling time of cells was estimated by dividing the cell number by the culture time, and the cisplatin‐resistance effect was detected by using a cell counting kit‐8 (CCK‐8) assay kit (CK04‐01; DOJINDO). For cisplatin resistance analysis, the cells were seeded into 96‐well plates at a density of 1000 cells/well. At the indicated times, 10 μl of the CCK‐8 solution was then added to each well followed by 2 h of culture. The absorbance at 450 and 600 nm was measured by using a Synergy H1 multifunctional microplate reader (Biotek).

### 2.4 Immunohistochemistry and immunofluorescence

Immunohistochemistry (IHC) and immunofluorescence (IF) were performed according to the procedures designed in previous studies.^17^ Briefly, cells and tumor tissues were fixed and permeabilized. Afterward, the cells were treated with the primary antibody, followed by incubation with the corresponding secondary antibody. The primary antibodies used in this study were anti‐Ki67 (Abcam; ab15580) and anti‐ITGB3 (Cell Signaling Technology; 13166), and the cell nuclei were labeled with 4′,6‐diamidino‐2‐phenylindole. IHC and IF images were obtained by using an FV3000 confocal fluorescence microscope (Olympus). To compare gene expression differences in different samples, the standard procedures described in previous studies^17^, ^18^ were used to perform quantitative immunofluorescence (QIF) analysis. The relative value of QIF was quantified using ImageJ software.

### 2.5 CRISPR/Cas9‐mediated ITGB3 knockout in 143B cells

To knock out ITGB3 in 143B cells, sgRNA (single guide RNA) sequences spanning exon 3 and exon 4 of ITGB3 were designed to decrease the potential off‐target points by using a web‐based tool (http://www.e‐crisp.org/E‐CRISP/). Then, the two sequences of each point were annealed and ligated into the vector pX459 (Addgene; #62988) to generate two plasmid constructs, pX459‐ITGB3‐gRNA1 and pX459‐ITGB3‐gRNA2. These two vectors were coelectroporated into 143B cells by using the Neon Transfection System (Thermo). After 24 h of cultivation, 1 μg/ml puromycin was added and selected for 48 h, and then the electroporated cells were seeded on 10 cm dishes at a very low density of approximately 2000 cells/dish. On day 14, we manually picked approximately 100 puromycin‐resistant colonies into 96‐well plates and then expanded them separately. Genomic DNA was purified from these picked cell colonies, and the homozygous mutant cells were confirmed via PCR amplification and Sanger sequencing by using specific primers that spanned the knockout fragments. The oligo sequences of the two gRNAs and the detected primers were as follows:

ITGB3‐gRNA1: TCACTCAAGTCAGTCCCCAG

ITGB3‐gRNA2: GGTGAGCTTTCGCATCTGGG

ITGB3‐KO‐F: ATAGCAGGGGTTTTCGAGGG

ITGB3‐KO‐R: GCCATAGCTCTGATTGCTGG

### 2.6 Real‐time quantitative reverse transcription PCR

Total RNA was purified using a TRIzol reagent (1–5 × 10^6^ cells/1 ml TRIzol) according to the manufacturer's procedure (Invitrogen). One microgram of total RNA was reverse transcribed into first strand cDNA with the HiScript^III^ first Strand cDNA Synthesis Kit (Vazyme). Then, real‐time quantitative reverse transcription PCR (RT–qPCR) procedures were conducted in triplicate using Taq Pro Universal SYBR qPCR Master Mix (Vazyme). GAPDH was used as a loading control for each gene, and comparative CT analysis was used to quantify the gene expression. At least three experiments were repeated. The primers used in this study were as follows:

hITG‐E6‐F: CCTAATGACGGGCAGTGTCA

hITG‐E9‐R: TCACGCACTTCCAGCTCTAC

hITG‐E5‐F: AGCAGAGTGTGTCACGGAAC

hITG‐E7‐R: TCAGTCATCAGCCCCAAAGAG

### 2.7 Tumor xenotransplantation in vivo

In the subcutaneous tumor xenotransplantation experiment, two cell lines, 143B and ITGB3‐KO cells, were washed and resuspended in phosphate‐buffered saline, and 1 × 10^6^ cells (100 μl) were subcutaneously injected into 6‐week‐old NOD/SCID mice (Charles River). From the seventh day of inoculation, they were treated with a control solution (normal saline) and cisplatin (4 mg/kg). All groups received their own treatments every 3 days for a total of seven treatments. The long (*l*) and short (*s*) diameters of the tumor were measured weekly, and the tumor volume was calculated according to the following formula: volume = (*l* × *s* ^2^)/2. Twenty‐eight days after cell injection, the mice were euthanized, and the tumors were collected for subsequent assays. All animal care and mouse testing procedures were approved by the Institutional Animal Care and Use Committee at Hospital (T.C.M.) Affiliated to Southwest Medical University.

### 2.8 RNA‐Seq and analysis

All RNA‐sequencing (RNA‐Seq) reads of 143B and ITGB3‐KO were mapped to the human genome GRCh38 (Hg38) using Bowtie2 (version 2.2.4). The read counts per gene were detected by using feature counts (Version 1.6.4), and differentially expressed gene analysis was constructed by using DESeq2.^19^ The Venn diagram was created using the Venny online tool (http://bioinfogp.cnb.csic.es/tools/venny). Kyoto Encyclopedia of Genes and Genomes (KEGG) pathway enrichment analysis was performed using the Cytoscape software (Cytoscape 3.9.1) STRING module (Search Tool for the Retrieval of Interacting Genes/Proteins version 10.0).

## 3 RESULTS

### 3.1 ITGB3 stimulated proliferation in osteosarcoma carcinoma

**FIGURE 1:**
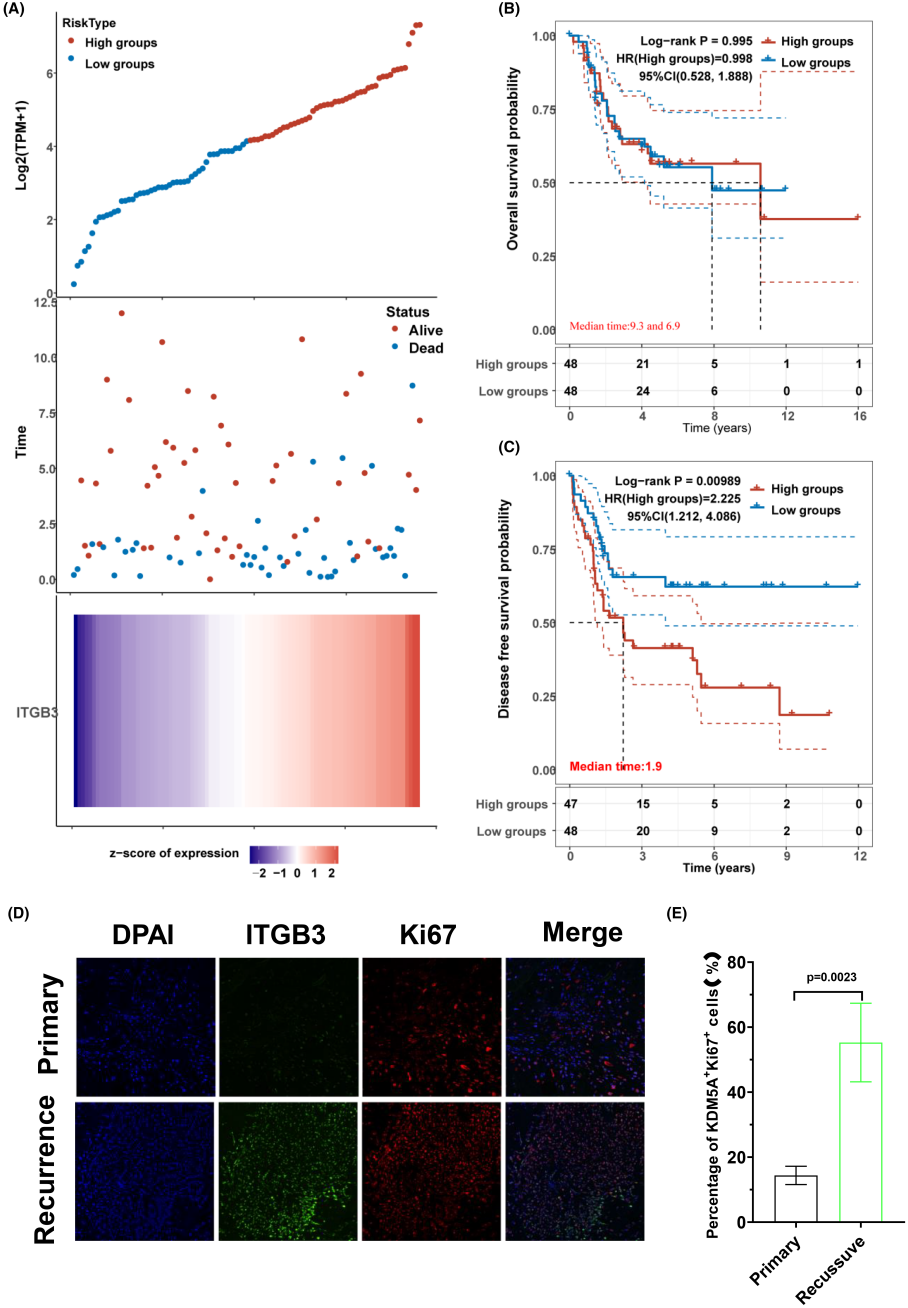
ITGB3 overexpression was associated with disease‐free survival and stimulated proliferation in osteosarcoma tissues. (A) Prognostic analysis of the ITGB3 signature in the TARGET datasets, where the top represents the scatter diagram of the gene expression of the median risk score from low to high and divides the patients into low‐risk (blue) and high‐risk (red) groups. The middle panel represents the scatter plot distribution of survival status and times responding to the expression of ITGB3 in different patients' tumor samples. At the bottom, the heatmap of the expression of ITGB3 is displayed. All the *x*‐axes of the top, middle, and bottom of the three figures represent the 95 patients' tumor samples, and the order was consistent. (B) Kaplan–Meier curve showing OS in the ITGB3 high‐expression and low‐expression groups. Among them, different groups were tested by the log‐rank. HR (high EXP) represents the risk coefficient of the high‐expression groups compared with the low‐expression groups. If HR > 1, this gene is a risk factor, and if HR < 1, this gene is a protective factor. The 95% CL represents the HR confidence interval; the median time represents the corresponding time (median survival time) of the high‐expression and low‐expression groups, and the unit is years. (C) Kaplan–Meier curve revealing the DFS in the ITGB3 high‐expression and low‐expression groups. The patients were grouped as shown in (B). (D) IHC staining was used to assess endogenous Ki67 and ITGB3 expression in paired primary and recurrent osteosarcoma tissues. IHC images were obtained by using a standard confocal fluorescence microscope (FV3000). Scale bar: 50 μm. (E) Histogram graph revealing the ratio of double‐positive ITGB3^+^/Ki67^+^ cells in primary and recurrent osteosarcoma samples. Student's *t* test was used to calculate the *p*‐value. CI, confidence interval; DFS, disease‐free survival; HR, hazard ratio; IHC, immunohistochemistry; OS, overall survival.

The RNA‐Seq raw count data and related clinical information of ITGB3 were obtained from the TARGET dataset (Table S1). Then, the risk score of every patient was calculated, the median cut‐off point was obtained, and the patients were divided into a high‐risk group (*n* = 47) and a low‐risk group (*n* = 48) based on ITGB3 expression (Figure 1A). KM survival analysis with the auto‐select best cut‐off was performed and revealed no significant difference between the high‐risk group and the low‐risk group for OS time (Figure 1B), but osteosarcoma patients with higher expression of ITGB3 had a shorter DFS time (Figure 1C). Since ITGB3 has an obvious causal or prognostic effect on osteosarcoma recurrence, we collected 18 paired osteosarcoma tissues (primary: *n* = 9, recurrence: *n* = 9) and detected the expression level of ITGB3 by IHC staining assays (Figure 1D). According to the expression level evaluation, ITGB3 was markedly overexpressed in recurrence tissues compared with primary tissues. Next, we assessed the cell division marker Ki67, which can indicate tumor cell growth, to confirm whether increased ITGB3 expression was associated with tumor cell proliferation (Figure 1D). The ratios of ITGB3‐positive (ITGB3^+^), Ki67‐positive (Ki67^+^), and ITGB3^+^/Ki67^+^ double‐positive (Figure 1E) osteosarcoma cells in the recurrence groups were significantly higher than those in the primary groups, which was in agreement with our prediction. Based on the above results, the increased expression of ITGB3 may facilitate cell proliferation and promote human osteosarcoma growth.

### 3.2 Knockout of ITGB3 reduced osteosarcoma cell proliferation

**FIGURE 2:**
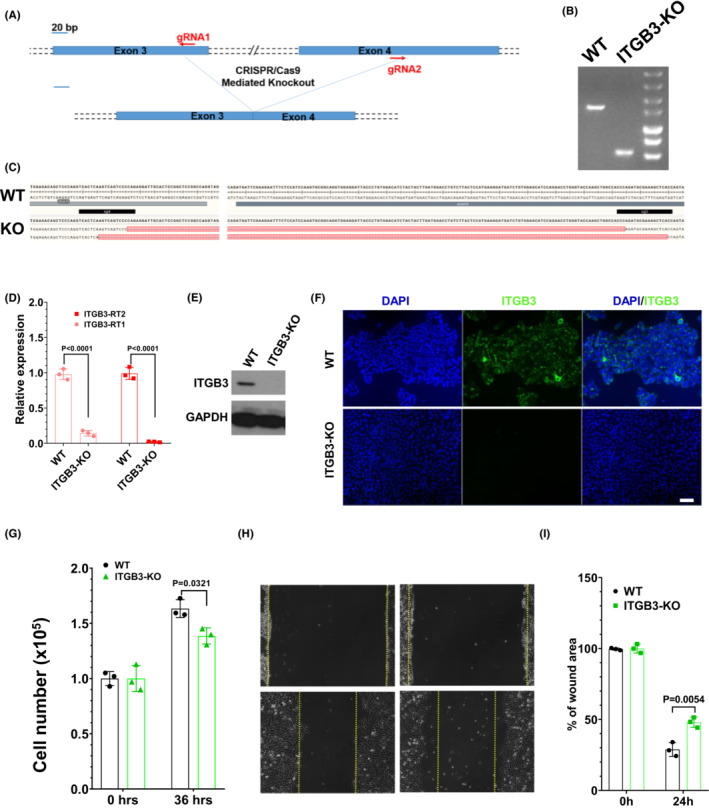
Knockout of ITGB3 reduced osteosarcoma cell proliferation. (A) Strategy to generate ITGB3‐KO cells by using the CRISPR/Cas9 system in the 143B cell line. In the upper panel, the exon information is shown. The target site and sgRNA sequences of exons 3 and 4 on ITGB3 are labeled in red. (B) Identification of the band of ITGB3^−/−^ (ITGB3‐KO) cells via gel electrophoresis of genomic PCR products. WT: wild‐type 143B cells. (C) Both alleles of targeted sequences were detected by Sanger sequencing in one mutant clone. A 28‐bp exon 3 and 140‐bp exon 4 were excised in one allele, and 33‐bp exon 3 and 138‐bp exon 4 were excised in another allele. (D) The expression levels of ITGB3 were detected by real‐time quantitative reverse transcription PCR analysis in the mutant ITGB3‐KO cell line. The expression levels of ITGB3 were normalized to those of GAPDH. Two paired primers and three replicated experiments were utilized, and the *p*‐values were calculated by using Student's *t* test. (E) Western blot analysis was used to detect the expression of ITGB3 in WT and ITGB3‐KO cell lines. GAPDH was used as an internal loading control. (F) The ITGB3 knockout efficiency in ITGB3‐KO cells was assessed by IF staining. 4′,6‐diamidino‐2‐phenylindole staining served as the internal loading control. (G) The number of ITGB3‐KO and 143B cells was counted on the indicated days in culture to evaluate the proliferation rate. (H) The scratch wound‐healing experiments showed the scratch region of 143B and ITGB3‐KO cells 24 h later. (I) Analysis of the percentage of the scratch area of 143B and ITGB3‐KO cells. The scratched area of wound healing was normalized to the initial area at *0 h*. All data in these figures were detected by three independent experiments, and the student's *t* test was used and is shown as the mean ± standard deviation. IF, immunofluorescence.

To further validate the potential functions of ITGB3 on osteosarcoma cell growth and migration in vitro, we used the CRISPR/Cas9‐mediated disruption system to generate the ITGB3‐knockout 143B cell line (Figure 2A). Genomic PCR products (Figure 2B) and Sanger sequencing (Figure 2C) confirmed that both alleles were mutated in ITGB3‐KO mice. RT–qPCR (Figure 2D), western blot (Figure 2E), and IF (Figure 2E) assays were used to detect ITGB3 expression and to clarify the knockout efficiency in ITGB3‐KO mutant cell lines. The growth of ITGB3 knockout 143B cells was markedly inhibited (Figure 2F). Furthermore, to determine whether ITGB3 affected cell migration, scratch wound healing experiments were performed and revealed that the scratch wound areas in ITGB3‐KO cells were significantly greater than those in 143B cells, which indicated that ITGB3 may play important functions in osteosarcoma motility (Figure 2G,H). Collectively, these results indicated that ITGB3 promotes osteosarcoma cell proliferation in vitro.

### 3.3 ITGB3 reduces osteosarcoma cell sensitivity to cisplatin

**FIGURE 3:**
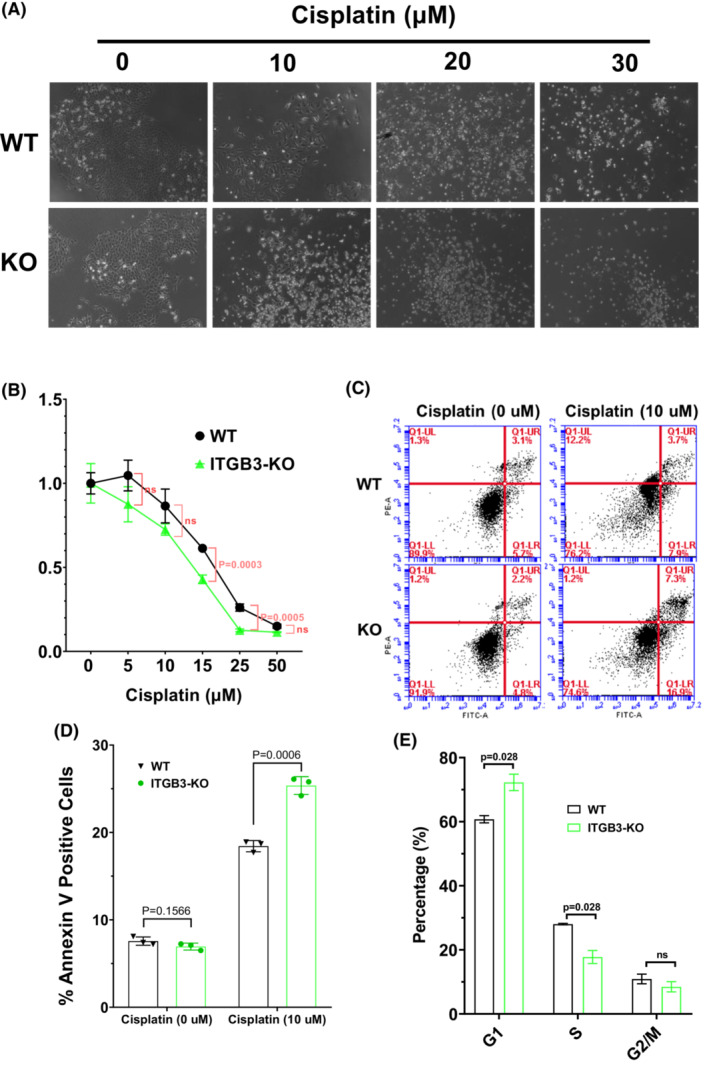
ITGB3 knockout reduced cisplatin resistance and promoted apoptosis. (A) Light microscopic images of 143B and ITGB3‐KO cells treated with different concentrations of cisplatin. (B) Graphical presentation of the viability of 143B and ITGB3‐KO cells treated with cisplatin. (C) Annexin V‐FITC/propidium iodide staining and FACS analysis were used to detect the percentage of apoptotic cells in 143B and ITGB‐KO cells with or without cisplatin treatment for 24 h. (D) The bar graph shows the percentage of Annexin V‐positive apoptotic cells between WT and ITGB3‐KO cells. WT: 143B cells. (E) The percentage of each cell phase among WT and ITGB3‐KO cells is shown in a histogram chart. All data were detected by three independent experiments, and Student's *t* test was used and is shown as the mean ± standard deviation. ns, not significant.

Notably, ITGB3 knockout decreased cell viability after treatment with 10, 20, and 30 μM cisplatin (Figure 3A). We then tested the functions of ITGB3 in the cisplatin resistance of osteosarcoma cells by using CCK‐8 assays and 143B cell knockout for ITGB3. ITGB3 knockout inhibited the viability of 143B cells at 24 h after treatment with 15 and 25 μM cisplatin (Figure 3B). We also analyzed the functions of ITGB3 in cisplatin‐induced apoptosis. ITGB3 KO significantly increased the rate of cisplatin‐induced apoptosis (25.3% vs. 12.86%, *p* = 0.0006) (Figure 3C,D). The cell cycle was analyzed by flow cytometry, which showed a decreased S‐phase ratio and an increased G1‐phase ratio after ITGB3 knockout. These results revealed that ITGB3 affects cell cycle progression (Figure 3E) and that the knockout of ITGB3 was negatively related to the cisplatin resistance of osteosarcoma cells in vitro. To summarize, ITGB3 expression was positively related to increased cisplatin resistance and decreased apoptosis in osteosarcoma progression.

### 3.4 ITGB3 knockout reduced tumor cisplatin resistance in vivo

**FIGURE 4:**
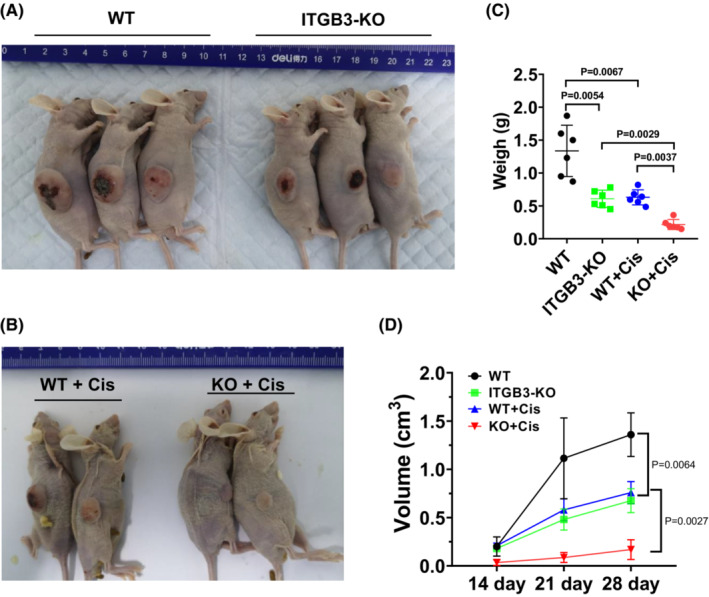
ITGB3 knockout reduced cisplatin resistance in vivo. (A) 143B and ITGB3‐KO cells were injected subcutaneously into the right side of the abdomen of NOD/SCID mice (1 × 10^6^ cells/site, *n* = 6). On *day 28*, the xenograft tumor‐bearing mice were anesthetized, sacrificed, and photographed. Scale bar: 1 cm. A representative picture of the tumor is shown in this figure, and other tumor samples are shown in Figure S1. (B) NOD/SCID mice were inoculated with 143B and ITGB3‐KO cells (1 × 10^6^ cells/site, *n* = 6). On *day 7*, treatment was initiated. In the cisplatin group, cisplatin (4 mg/kg) in 100 μl of 0.9% saline solution was administered via intraperitoneal injection. In the control group, the control solution (200 μl of 0.9% saline) was injected. All groups were administered their own treatments every 3 days for a total of seven times. A representative picture of the tumor is shown in this figure, and other tumor samples are shown in Figure S1. (C) The tumor volume was observed and measured once a week in WT and ITGB3‐KO mice with or without cisplatin treatment (each group, *n* = 6). (D) The weights of the tumors were weighed when the mice were sacrificed (each group, *n* = 6).

To study whether ITGB3 can affect osteosarcoma cisplatin resistance in vivo, we subcutaneously injected ITGB3‐KO and 143B cells into NOD/SCID mice (*day 0*). From the seventh day of inoculation, they were treated with a control solution (normal saline) and cisplatin (4 mg/kg). All groups received their own treatments every 3 days for a total of seven treatments. The long and short diameters of the tumors were measured once a week. At *day 28*, the tumor tissues were isolated from the tumor‐bearing NOD/SCID mice (Figure 4A,B). When comparing the ITGB3‐KO group and the 143B group, the tumor weights were decreased (Figure 4C), and tumor volumes were diminished (Figure 4D), which indicated that ITGB3 knockout could suppress tumor growth in vivo. Further analysis revealed that treatment with cisplatin significantly decreased the weights (Figure 4C) and volumes (Figure 4D) of the tumors derived from the ITGB3‐KO cell line, which was in accordance with our in vitro data. Therefore, these findings support that ITGB3 knockout could potentiate osteosarcoma sensitivity to cisplatin in vivo.

### 3.5 Transcriptome signatures associated with ITGB3 in osteosarcoma

**FIGURE 5:**
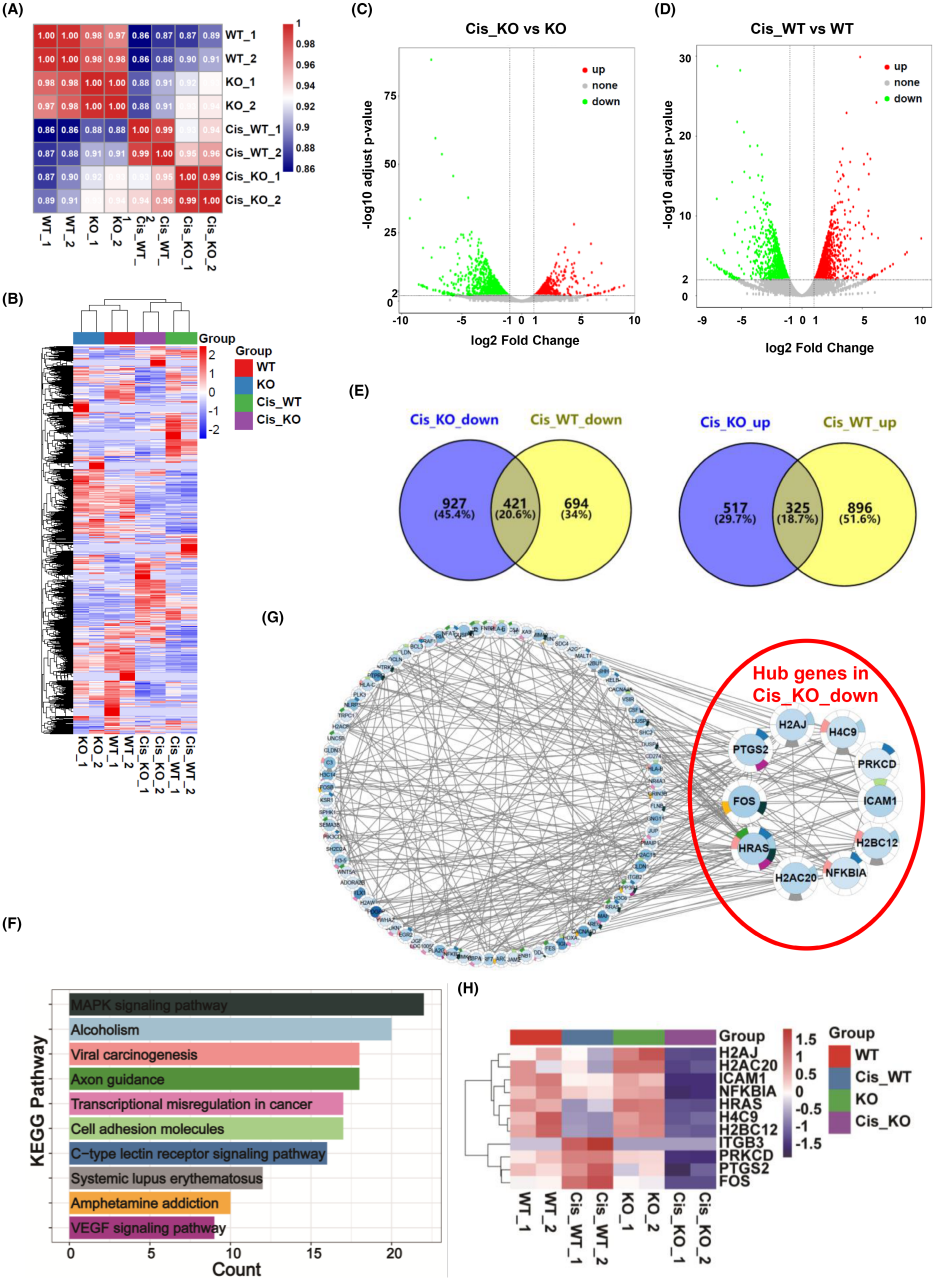
The transcriptome of ITGB3‐KO cells. (A) Pearson correlation heatmap associating WT, ITGB3‐KO, Cis_WT, and Cis_KO. Colors from blue to red indicate weak to strong correlations. KO: ITGB3‐KO cell line; WT: 143B cell line. (B) A heatmap shows the different levels of RNA expression in WT, KO, Cis_WT, and Cis_KO. (C) A volcano plot displaying the DEGs when comparing Cis_KO to KO. Red represents the upregulated genes (log2FoldChange >1, −log10 adjusted *p* value >2) in Cis_KO compared with KO. Green indicates the downregulated genes (log2FoldChange <−1, −log10 adjusted *p* value >2) in Cis_KO compared with KO. Gray represents nonsignificant DEGs. (D) A volcano plot displaying the DEGs when comparing Cis_WT to WT. Red represents the upregulated genes (log2FoldChange >1, −log10 adjusted *p* value >2) in Cis_WT compared with WT. Green indicates downregulated genes (log2FoldChange <−1, −log10 adjust *p* value >2) in Cis_WT compared with WT. Gray represents nonsignificant DEGs. (E) The Venn diagram on the left shows the overlap of genes between Cis_KO_down and Cis_WT_down, and the right shows the overlap of genes between Cis_KO_up and Cis_WT_up. (F) The enriched KEGG pathways of Cis_KO_down were generated based on the STRING database in Cytoscape. (G) The 10 hub genes and real genes of the KEGG pathways of Cis_KO_down are shown in (F) with the MCODE algorithm in Cytoscape. (H) Heatmap showing the expression of ITGB3 and 10 hub genes in WT, KO, Cis_WT, and Cis_KO. The raw data of (B–E) are shown in Table S2, and the raw data of (F) are shown in Table S3. DEG, differentially expressed gene; KEGG, Kyoto Encyclopedia of Genes and Genomes.

To elucidate the underlying mechanism of the anti‐cisplatin effect of ITGB3 in osteosarcoma, we collected total RNA and performed transcriptome analysis on 143B (WT), ITGB3‐KO (KO), cisplatin‐treated 143B (Cis_WT) and ITGB3‐KO (Cis_KO) cells. The correlation heatmap (Figure 5A,B) and PCA (Figure S1) revealed that KO and WT showed small differences, while Cis_KO and Cis_WT showed significant differences. There was a different expression pattern when comparing Cis_WT with WT cells and Cis_KO with WT cells, which was demonstrated via a volcano plot analysis (Figure 5C,D). There were 1221 upregulated genes (Cis_WT_up) and 1115 downregulated genes (Cis_WT_down) in Cis_WT compared with WT, and there were 842 upregulated genes (Cis_KO_up) and 1348 downregulated genes (Cis_KO_down) in Cis_KO compared with KO (Figure 5C,D; Table S2). The number of up and downregulated genes between the Cis_KO and Cis_WT groups is shown (Figure 5E; Table S2). To investigate the mechanism by which ITGB3 exerts cisplatin resistance in osteosarcoma, we analyzed the genes that were only upregulated and downregulated in the Cis_KO group using the computational tool Cytoscape and STRING enrichment analysis. It is interesting to note that KEGG pathway enrichment was observed only in the downregulated group (Figure 5F; Table S3). Among these enrichments, some signaling pathways were commonly related to cisplatin resistance in osteosarcomas, such as the mitogen‐activated protein kinase (MAPK) pathway^20^, ^21^, ^22^, ^23^, ^24^ and vascular endothelial growth factor (VEGF).^25^, ^26^, ^27^ Additionally, 10 hub genes were identified, and their associated signaling pathways were demonstrated (Figure 5G). These hub genes also appeared to play a regulatory role in reducing cisplatin resistance when ITGB3 was knocked out (Figure 5G). ICAM1, PTGS2, HRAS, and NFKBIA. Accordingly, these analyses suggested that ITGB3 may perform the functions of cisplatin resistance in osteosarcoma through these signaling pathways.

## 4 DISCUSSION

Long‐term clinical drug therapy may cause drug resistance, followed by osteosarcoma recurrence and poor clinical outcomes. Cisplatin, one of the most common first‐line chemotherapy drugs, is the most active drug for osteosarcoma treatment.^28^ Therefore, a better understanding of the underlying molecular signaling networks that regulate cisplatin resistance may represent more feasible options to effectively decrease drug resistance and improve patient OS. In the present work, ITGB3 was identified as a potential regulator of tumorigenicity and cisplatin resistance in osteosarcoma through public clinical dataset (TARGET database) analysis, clinical sample detection, and functional experimental model testing. First, using a clinical database and clinical samples, we found that ITGB3 was more highly expressed in recurrent osteosarcoma patients and was associated with tumor proliferation. Furthermore, the decreased osteosarcoma cell proliferation and migration ability in ITGB3 knockout osteosarcoma cells were also confirmed. The decreased cell growth was related to increased apoptosis and slowing cell cycle progression. We further discovered that ITGB3 had a positive correlation with cisplatin resistance through tumor xenografts in mice.

Next, through Cytoscape and STRING analysis of cisplatin‐treated or nontreated WT and ITGB3‐KO cell‐related RNA‐Seq data, we found that ITGB3 plays a role in promoting osteosarcoma progression through multiple signaling pathways. The combined analysis of hub genes and enriched signaling pathways (Figures S2–S4) revealed that hub genes were regulated by these signaling pathways, including the MAPK, VEGF, C‐type lectin receptor, and cell adhesion molecule signaling pathways. Among them, the key hub genes were ICAM1 (intercellular adhesion molecule 1), HRAS (Ras family small GTP binding protein H‐Ras), PTGS2 (prostaglandin‐endoperoxide synthase 2, also known as COX‐2), and FOS (FBJ osteosarcoma oncogene). Interleukin‐6 (IL‐6) activated the expression of ICAM‐1 and contributed to the migration of human osteosarcoma cells through integrin‐linked kinase (ILK)/Akt/AP‐1 pathways.^29^ Osteosarcoma cells colonize the lungs more quickly by upregulating ICAM1 expression in response to tumor‐derived IL‐6, which facilitates glycolytic metabolism in tumor cells by activating the MEK/ERK/HIF‐1α pathway.^30^ On the surface of the endothelium, cisplatin upregulates the expression of intercellular adhesion molecules, which causes increased infiltration of monocytes or leukocytes. Cisplatin upregulated endothelial ICAM‐1 expression through an NF‐κB‐dependent mechanism (31). HRAS belongs to the Ras oncogene family, whose members are associated with mammalian sarcoma retroviruses. In osteosarcoma cells, a dominant‐negative H‐RAS mutant (17N) partially suppressed ERK activation and delayed apoptosis induced by chelerythrine.^32^ Notably, gallium(III) complex 1, which has higher toxicity toward osteosarcoma cells grown than cisplatin, downregulates COX‐2 expression and kills osteosarcoma cells in a COX‐2‐dependent manner.^33^ ERK/JNK and c‐JUN/c‐FOS, as upstream activators of FOXP1, drive osteosarcoma development.^34^ FOS also efficiently initiates key stem cell proliferation, migration, division, and differentiation.^35^ FOS can regulate prognostic genes related to osteosarcoma metastasis.^36^ These results indicated that ICAM1, HRAS, PTGS2, and FOS, as downstream genes, may predict the potential of cisplatin treatment. Thus, targeted suppression of the MAPK and VEGF pathways in combination with cisplatin treatment may be a promising method for overcoming osteosarcoma chemoresistance. In conclusion, a thorough investigation of the molecular mechanism by which ITGB3 participates in osteosarcoma cisplatin resistance via the involvement of the signaling pathway should promote treatment effects.

Taken together, our data showed that ITGB3 may be a critical regulator of cisplatin resistance in osteosarcoma. Our results will contribute to a better understanding of the function and mechanism of ITGB3 in osteosarcoma cisplatin resistance and provide a novel therapeutic target to decrease cisplatin resistance and tumor recurrence in osteosarcoma patients.

## AUTHOR CONTRIBUTIONS

**Qian Li:** Data curation (equal); methodology (equal). **Guangyou Chen:** Conceptualization (equal); data curation (equal). **Huachai Jiang:** Data curation (equal); software (equal). **Haoping Dai:** Methodology (supporting). **Dongdong Li:** Data curation (supporting); software (supporting). **Kai Zhu:** Methodology (supporting). **Kaiquan Zhang:** Methodology (supporting). **Huarui Shen:** Funding acquisition (supporting); writing – original draft (equal). **Houping Xu:** Funding acquisition (equal); writing – original draft (equal); writing – review and editing (equal). **Sen Li:** Funding acquisition (lead); writing – review and editing (lead).

## CONFLICT OF INTEREST STATEMENT

All the authors declare no competing financial interests.

## Supporting information


Table S1.

Table S2.

Table S3.

Figure S1.
Figure S2.
Figure S3.
Figure S4.
Figure S5.
Figure S6.

## Data Availability

The data that supuport the finding of this study are available from the corresponding author (lisen_swmctcm@163.com) upon reasonable request. The data are not publicy available due to privacy restrictions.
