# Supplementary material for: ITGB3 promotes cisplatin resistance in osteosarcoma tumors
Source: Cancer Med. 2023 Feb 11;12(7):8452–63. doi: 10.1002/cam4.5585 (PMC10134362; doi:10.1002/cam4.5585)

Supplementary Figure 1

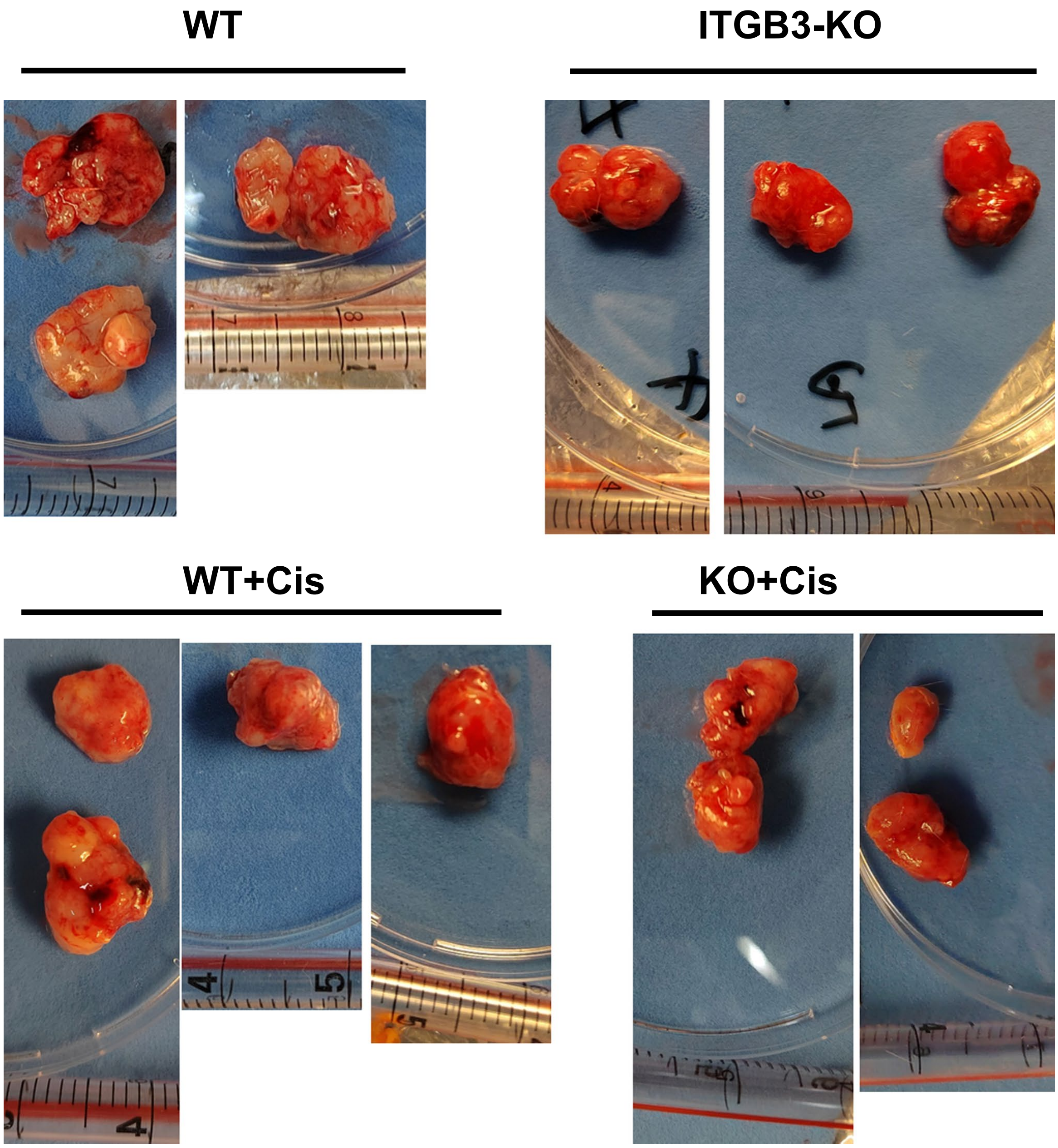

Supplementary Figure 2

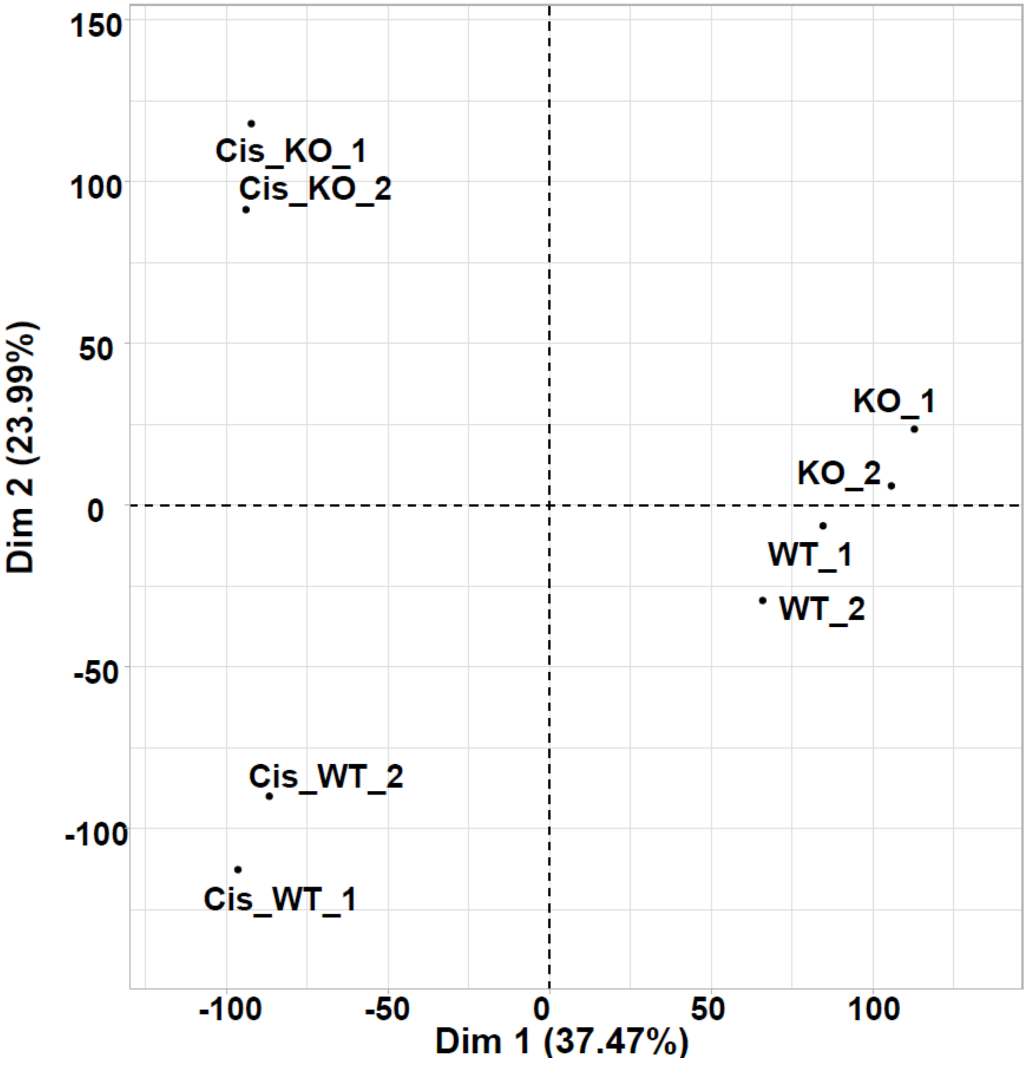

Supplementary Figure 3

Cell adhesion molecules

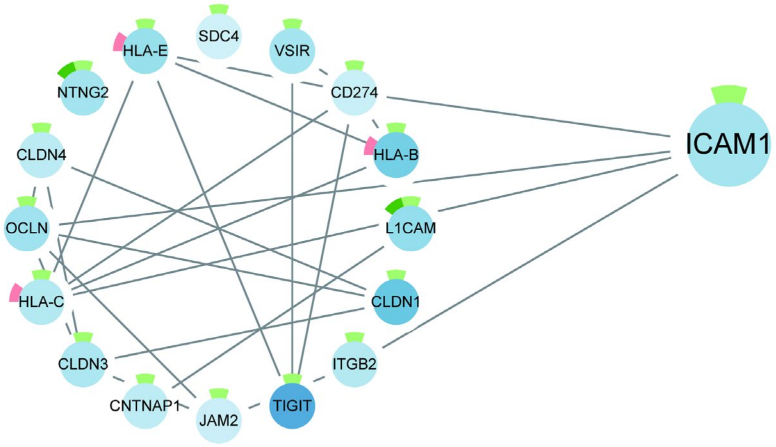

C-type lectin receptor signaling pathway

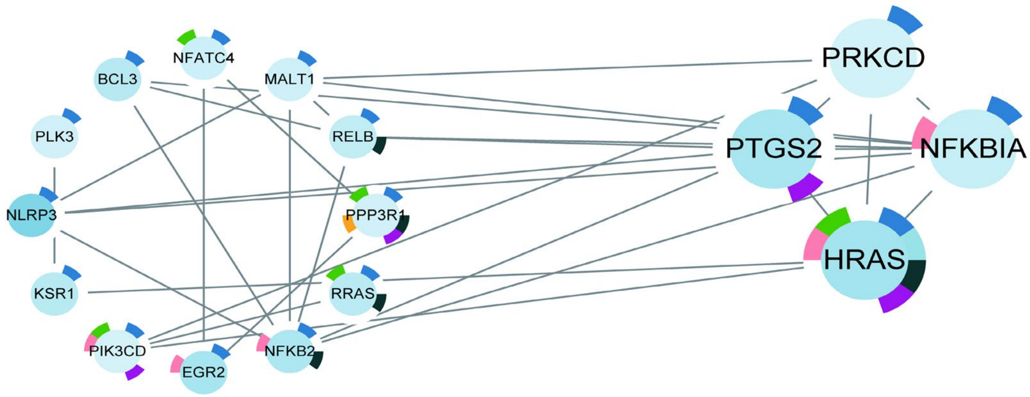

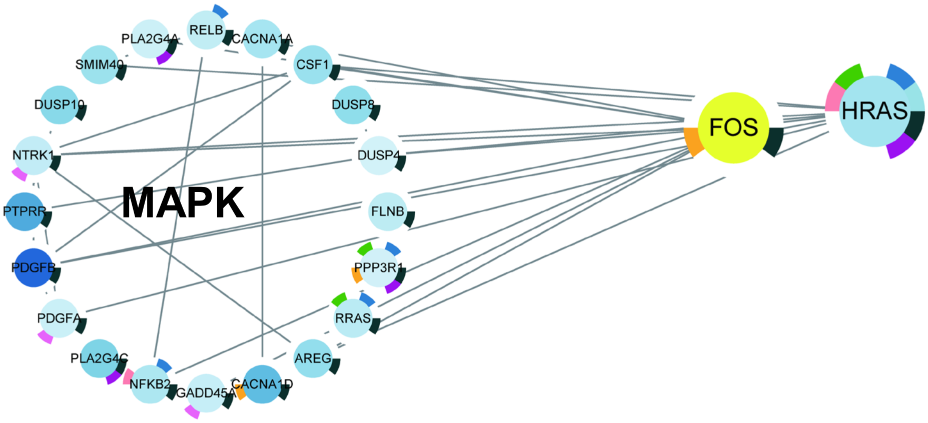

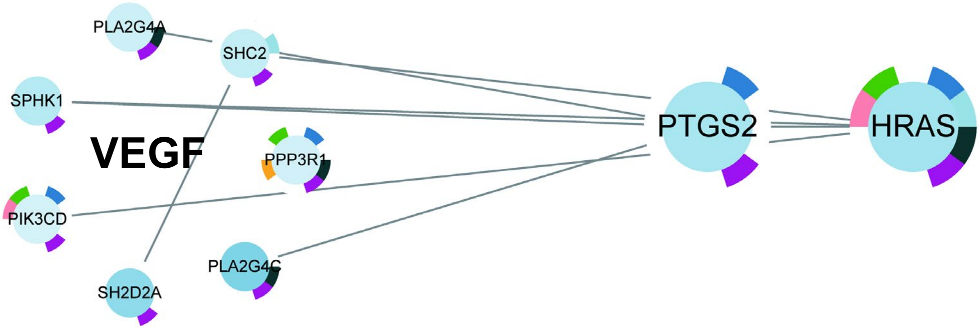

Supplementary Figure 4

Alcoholism

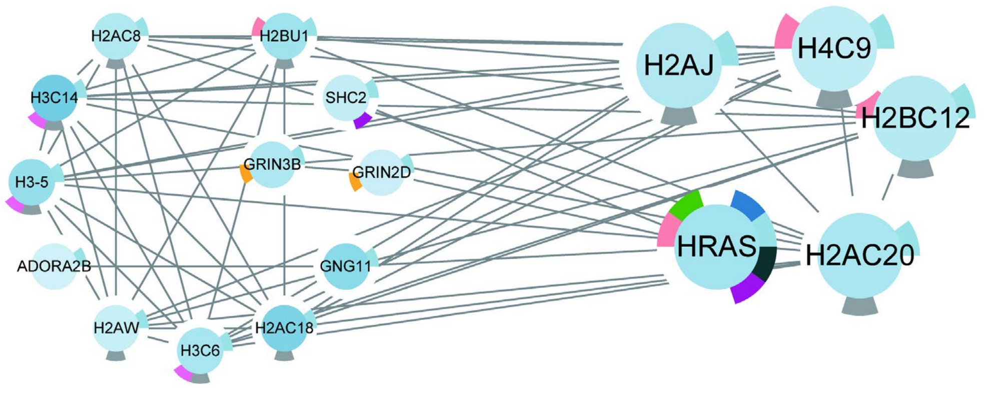

Amphetamine addiction

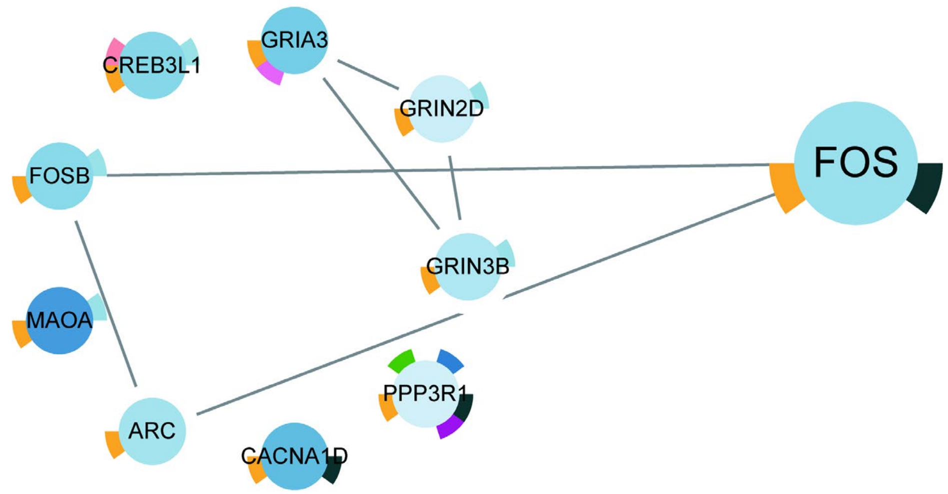

Axon guidance

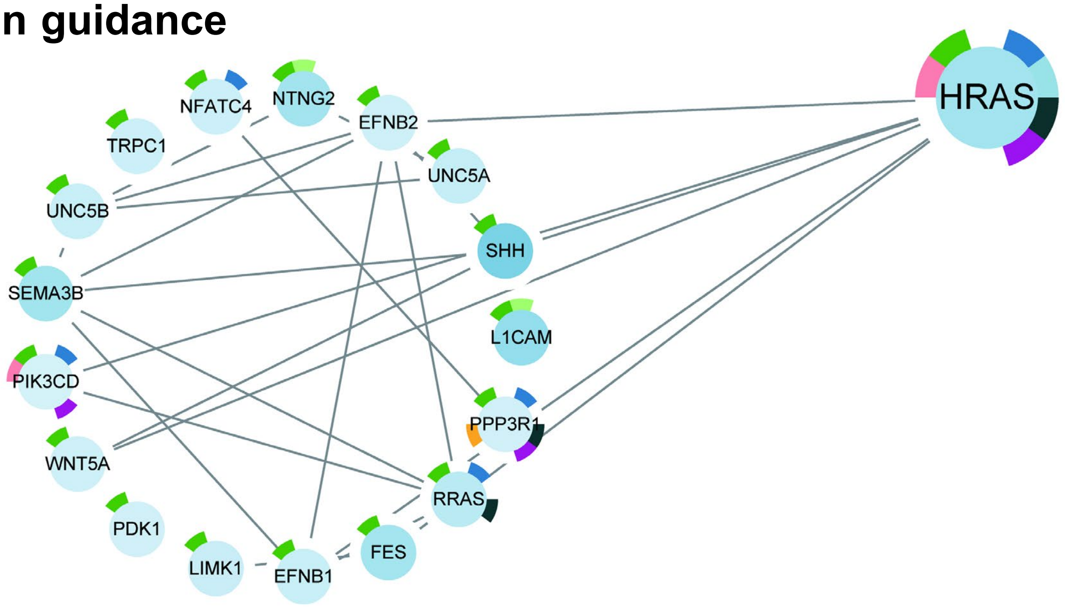

Supplementary Figure 5

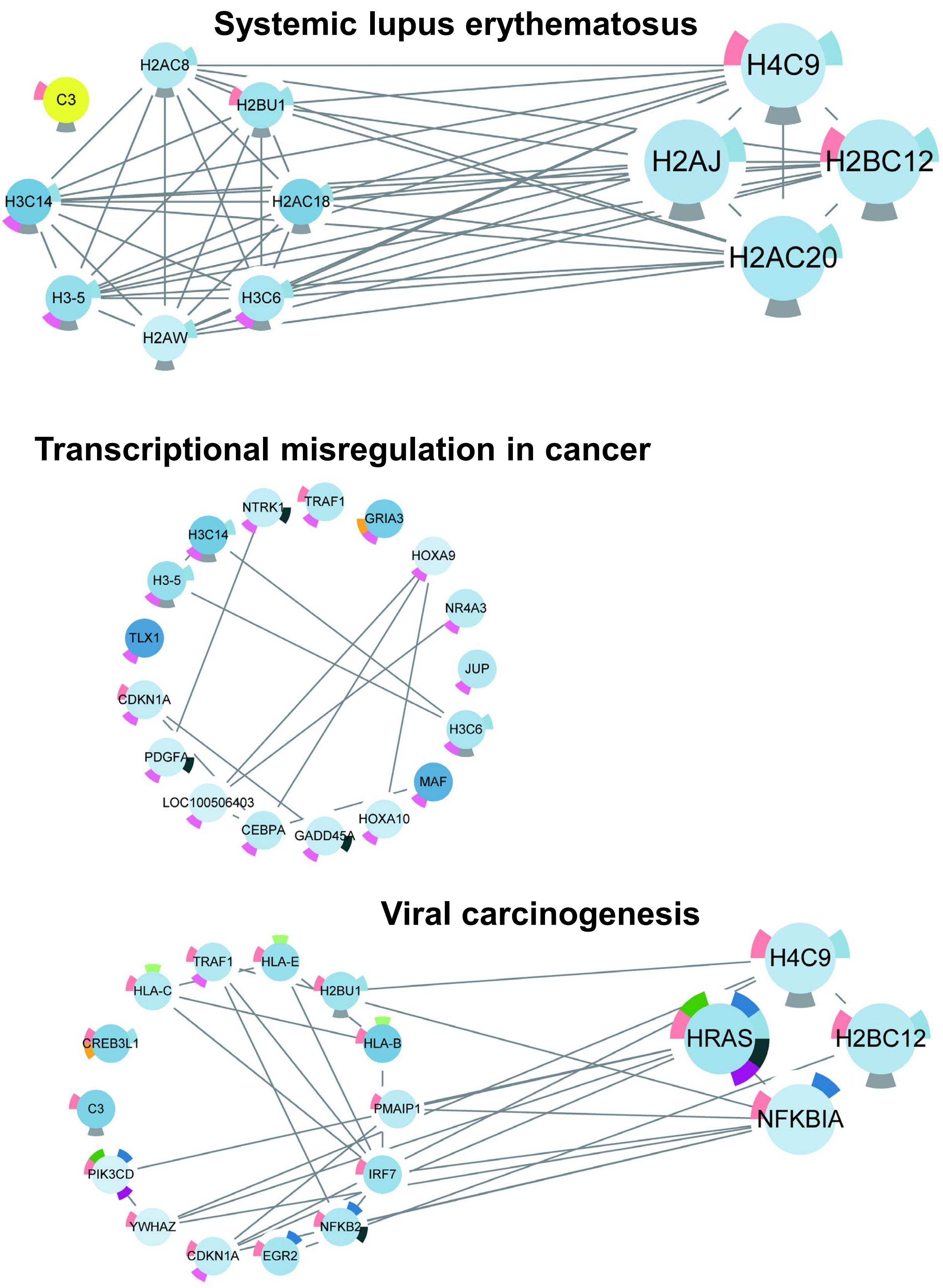

Supplementary Figure 6

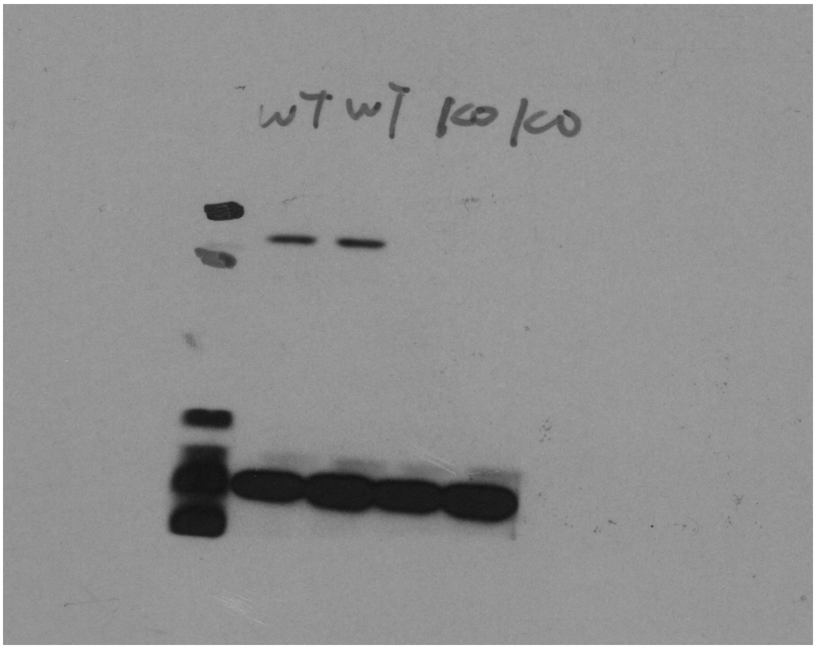

Supplement: Supplementary file 4 — Figure S1. Figure S2. Figure S3. Figure S4. Figure S5. Figure S6. [file CAM4-12-8452-s001.pdf]
